# Technical Pitfalls of Signal Truncation in Perfusion MRI of Glioblastoma

**DOI:** 10.3389/fneur.2016.00121

**Published:** 2016-08-02

**Authors:** Kelvin K. Wong, Steve H. Fung, Pamela Z. New, Stephen T. C. Wong

**Affiliations:** ^1^Department of Systems Medicine and Bioengineering, Houston Methodist Research Institute, Houston, TX, USA; ^2^Department of Radiology, Weill Cornell Medicine, Cornell University, New York, NY, USA; ^3^Department of Neurological Surgery, Weill Cornell Medicine, Cornell University, New York, NY, USA

**Keywords:** glioblastoma multiforme, perfusion MRI, gene therapy, MRI imaging, neuro-oncology

## Abstract

Dynamic susceptibility contrast (DSC) perfusion-weighted imaging (PWI) is widely used in clinical settings for the radiological diagnosis of brain tumor. The signal change in brain tissue in gradient echo-based DSC PWI is much higher than in spin echo-based DSC PWI. Due to its exquisite sensitivity, gradient echo-based sequence is the preferred method for imaging of all tumors except those near the base of the skull. However, high sensitivity also comes with a dynamic range problem. It is not unusual for blood volume to increase in gene-mediated cytotoxic immunotherapy-treated glioblastoma patients. The increase of fractional blood volume sometimes saturates the MRI signal during first-pass contrast bolus arrival and presents signal truncation artifacts of various degrees in the tumor when a significant amount of blood exists in the image pixels. It presents a hidden challenge in PWI, as this signal floor can be either close to noise level or just above and can go no lower. This signal truncation in the signal intensity time course is a significant issue that deserves attention in DSC PWI. In this paper, we demonstrate that relative cerebral blood volume and relative cerebral blood flow (rCBF) are underestimated due to signal truncation in DSC perfusion, in glioblastoma patients. We propose the use of second-pass tissue residue function in rCBF calculation using least-absolute-deviation deconvolution to avoid the underestimation problem.

## Introduction

Dynamic susceptibility contrast (DSC) perfusion-weighted imaging (PWI) is widely used in the radiological diagnosis of brain tumors in addition to contrast-enhanced MRI and morphological MRI. In therapeutic monitoring of brain tumors, the long standing problem is how to differentiate tumor recurrence and pseudoprogression after chemoradiation as all of these scenarios will show contrast enhancement in MRI. The literature regarding the use of perfusion to differentiate these confusing scenarios has been shown to be very useful (1–5). Machine learning-based method in the differentiation showed that perfusion measurements, such as relative cerebral blood volume (rCBV) and relative cerebral blood flow, are indeed more useful than contrast enhancement (2). Dynamic contrast enhancement (DCE) permeability mapping also showed some promise in the area (6), though it was not directly compared to DSC perfusion. The variable success in application of DSC perfusion in distinguishing progression from pseudoprogression may rest on the details of how the perfusion studies are post-processed, leading to the difficulties in comparing results across studies (7). Standardization of post-processing algorithms along with injection and imaging protocols are important issues to application of DSC perfusion in brain tumors diagnosis.

Among the three cerebral hemodynamics parameters derived from DSC perfusion: rCBV, relative cerebral blood flow (rCBF), and mean transit time (MTT), rCBV is the most stable, as it is computed by the area under the curve of the contrast concentration time course normalized to the area under the arterial input function (AIF). There are various methods, including deconvolution-based methods (8–10) and the tissue residue function model-based method (11), to derive rCBF and MTT. Due to the impulse response nature of the tissue residue function, which has a sharp rising edge at contrast arrival, and the limited temporal resolution, all of these methods will underestimate the peak of the tissue residue function, which leads to severe underestimation of rCBF at high flow rate compared to lower flow rate (8–10). One way to resolve this issue is to have an accurate estimation of bolus arrival time (12), removing one parameter from the deconvolution of the tissue residue function, as a small error in the estimation of bolus arrival time can change the rCBF estimates significantly (13).

In clinical settings, the signal change in brain tissue in gradient echo DSC PWI is much higher than spin echo DSC PWI. Due to this exquisite sensitivity of gradient echo sequence, a lower contrast dose is required. Typically, spin echo DSC PWI is about half the sensitivity of gradient echo DSC PWI, with the former requiring injecting 2× the volume of contrast agent at the same concentration. Therefore, DSC PWI is a preferred method except at the base of the skull or other locations where the air–tissue interface causes significant signal dropout.

However, high sensitivity also comes with the dynamic range problem. Typical white matter and gray matter have about 2.5–5% fractional blood volume, and it is typical to tune the imaging parameters to obtain 30–60% signal drop in different brain tissues. Therefore, it is very common for the tumor signal intensity time course in gradient echo DSC PWI to drop to a noise level in pixels with a significant amount of blood volume, especially in pixels with rich venous blood or in the case of intratumoral hemorrhage. It presents a hidden challenge in PWI as the signal intensity time course can drop to a signal floor or noise floor level. In the first scenario, the average signal from the pixel is significantly above noise level because part of the pixel is occupied by a blood vessel. This signal truncation in the signal intensity time course is a significant issue that deserves attention in DSC PWI. In this paper, we demonstrate the effects of signal truncation in DSC perfusion studies of glioblastoma patients undergoing gene-mediated cytotoxic immunotherapy studies. The impact on CBF estimation is determined by the recirculating second pass of the tissue residue function when contrast leakage correction is used.

## Materials and Methods

### Theory

To measure cerebral hemodynamic parameters, DSC perfusion image pixel intensity needs to be converted to the concentration of contrast in the tissue. The relationship between signal intensity *S*(t) and concentration *C*(t) is defined by:

(1) $$S\left(t\right)=S_{0}e^{-\mathrm{kC}\left(t\right)\times \mathrm{TE}}$$

where *S*_0_ is the image intensity before contrast injection, *k* is a constant, and *TE* is the echo time. The tissue contrast concentration *C*(t) can be expressed as a convolution of the AIF with a tissue residue function *R*(*t*) multiplied by CBF:

(2) $$C(t)=F_{t}\int_{0}^{t}\mathrm{AIF}(\tau )R(t-\tau )\mathrm{d\tau}$$

(3) $$\text{MTT}=\int_{0}^{\infty }t\times h\left(t\right)\mathrm{dt}$$

(4) $$R\left(t\right)=1-\int_{0}^{t}h\left(t\right)\mathrm{dt}$$

where *h*(*t*) is the transport function or the probability distribution of transit time in the tissue and *F*_t_ is the cerebral blood flow (CBF). The tissue contrast concentration *C*(*t*) can be expressed as the convolution of the AIF, *C*_a_(*t*), with a residue function *R*(*t*) multiplied by CBF.

(5) $$\left[\begin{array}{c} C(t_{0})\\ C(t_{1})\\ \vdots \\ C(t_{N-1}) \end{array}\right]=\mathrm{\Delta t}\left[\begin{array}{cccc} C_{a}(t_{0}) & 0 & \cdots & 0\\ C_{a}(t_{1}) & C_{a}(t_{0}) & \ldots & 0\\ \vdots & \vdots & \ddots & \vdots \\ C_{a}(t_{N-1}) & C_{a}(t_{N-2}) & \cdots & C_{a}(t_{0}) \end{array}\right]\times \left[\begin{array}{c} R(t_{0})\\ R(t_{1})\\ \vdots \\ R(t_{N-1}) \end{array}\right]F_{\text{t}}$$

which can be simplified to *c* = *A*⋅*r* and CBF can be estimated as the maximum value of *r*.

The CBF rate can be estimated by a perfusion deconvolution algorithm using least-absolute-deviation (LAD) regularization (10), which allows more accurate estimation at high blood volume and high flow rate. LAD regularization can be achieved by minimizing the following functional:

(6) $$r_{\lambda }=\min{\;}_{r}\left\{{\left\|\mathrm{Ar}-c\right\|}_{1}+\lambda {\left\|\mathrm{Lr}\right\|}_{1}\right\}$$

where L1 norm is defined as $\left|\right|x{\left|\right|}_{1}=\sum_{i=1}^{n}\left|x_{i}\right|$; the regularization function *L* is chosen as a first order difference operator and λ is the regularization parameter. The minimization problem is formulated as a linear programing problem and solved iteratively by the interior point method (14).

Note that the ideal tissue residue function *R*(*t*) in the absence of recirculation is by definition a monotonic decay function, such as exponential, boxcar, or triangular functions (10). In the presence of second-pass recirculation, the measured tissue residue function *R*_m_(*t*) is a summation of the tissue residue function of the first pass and the second pass with a time-shift corresponding to the vascular delay (*V*_d_) and a Gaussian-like vascular dispersion function [*D*(*t*)] caused by the recirculation system.

(7) $$R_{\text{m}}\left(t\right)=R\left(t\right)+D\left(t\right)⊗R\left(t-V_{\text{d}}\right)$$

Assuming the first-pass tissue residue function does not extend beyond *V*_d_, which is a reasonable assumption in brain tissue, as *V*_d_ is at least 20 s in typical adult with normal cardiac output, rCBF can be determined as the peak of the second-pass tissue residue function, as the same dispersion function applies linearly on all pixels in tissue.

### Leakage Correction in Brain Tumor

Contrast leakage is expected to occur in most brain tumors and it is also one of the fundamental criteria to detect the spatial extent of brain tumors, clinically. Some brain tumors are non-enhancing though glioblastoma are mostly enhancing tumors with some non-enhancing components. In the presence of contrast leakage to parenchyma in strongly T2*-weighted PWI, the measured contrast concentration in tissue, *C*_m_(*t*), calculated using Eq. 1 can be effectively modeled as (15):

(8) $$C_{\text{m}}\left(t\right)=K_{1}C_{\text{tissue}}\left(t\right)+K_{2}\int_{0}^{t}C_{\text{tissue}}\left(t\right)$$

where *C*_tissue_(*t*) is the mean contrast concentration curve of normal brain tissue including both gray and white matter, *K*_2_ is a heuristic factor primarily determined by the rate of leakage, such as permeability surface area product, etc. The leakage corrected tissue contrast concentration *C*(*t*) is thus equal to *K*_1_*C*_tissue_(*t*).

### Clinical Cases

To demonstrate the signal truncation effects in rCBF measurements, longitudinal data from glioblastoma patients in gene-mediated cytotoxic immunotherapy studies (16) (www.ClinicalTrials.gov; ID: NCT00751270 and NCT00589875) were retrieved from the picture archiving system, retrospectively. The DSC perfusion study was conducted in a General Electric 1.5 T/3.0 T Signa Excite system using a 8-channel head coil. The imaging parameters of the 1.5 T protocol are: TR/TE: 1400–1700 ms/60 ms, 2D single-shot gradient-echo EPI sequence with 7 mm slice thickness with in-plane resolution of 1.875 mm and the 3.0 T protocol parameters are TR/TE:1600–2000 ms/40 ms, 2D single shot gradient-echo EPI sequence with 5 mm slice thickness with same in-plane resolution. Some cases were conducted with a power injector where 15 mL contrast agent is injected at 5 mL/s, intravenously. The study was approved by the institutional review board of the Houston Methodist Hospital.

## Results

**Figure 1:**
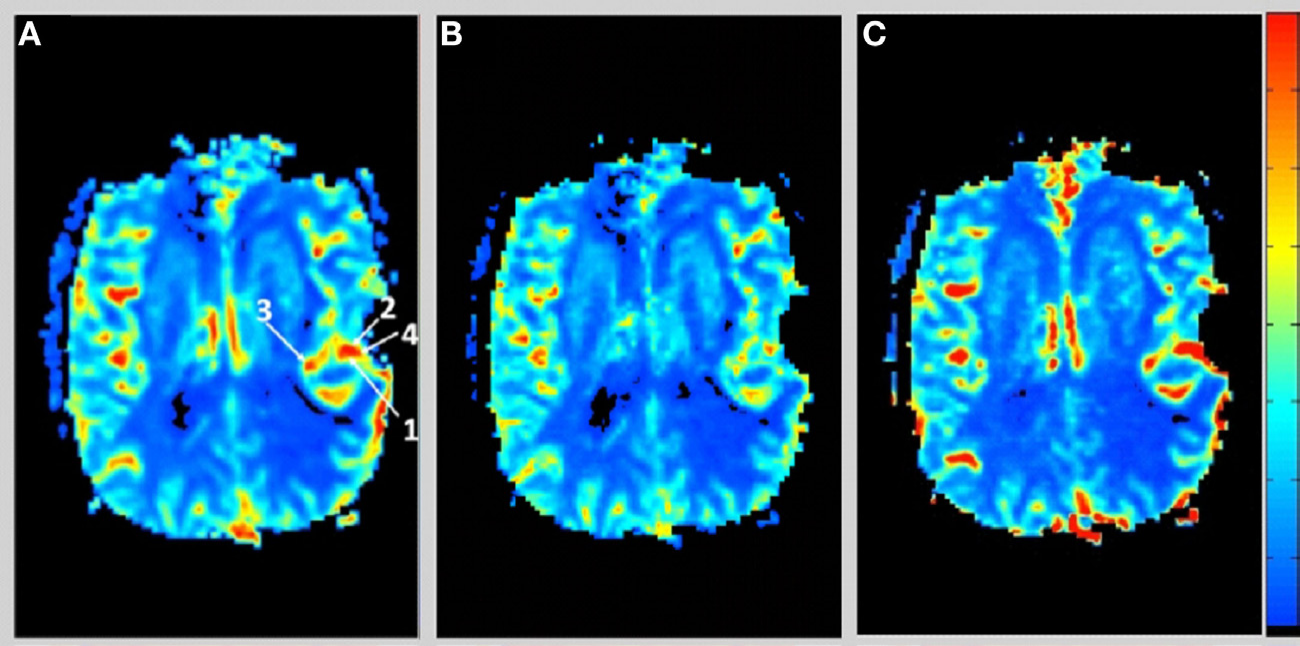
**(A)** Relative cerebral blood volume map generated from the leakage corrected data, **(B)** relative cerebral blood flow map determined by the peak of the first-pass tissue residue function, and **(C)** relative cerebral blood flow map determined by the peak of the second-pass tissue residue function. Note the tight coupling between blood volume and blood flow.

**Figure 2:**
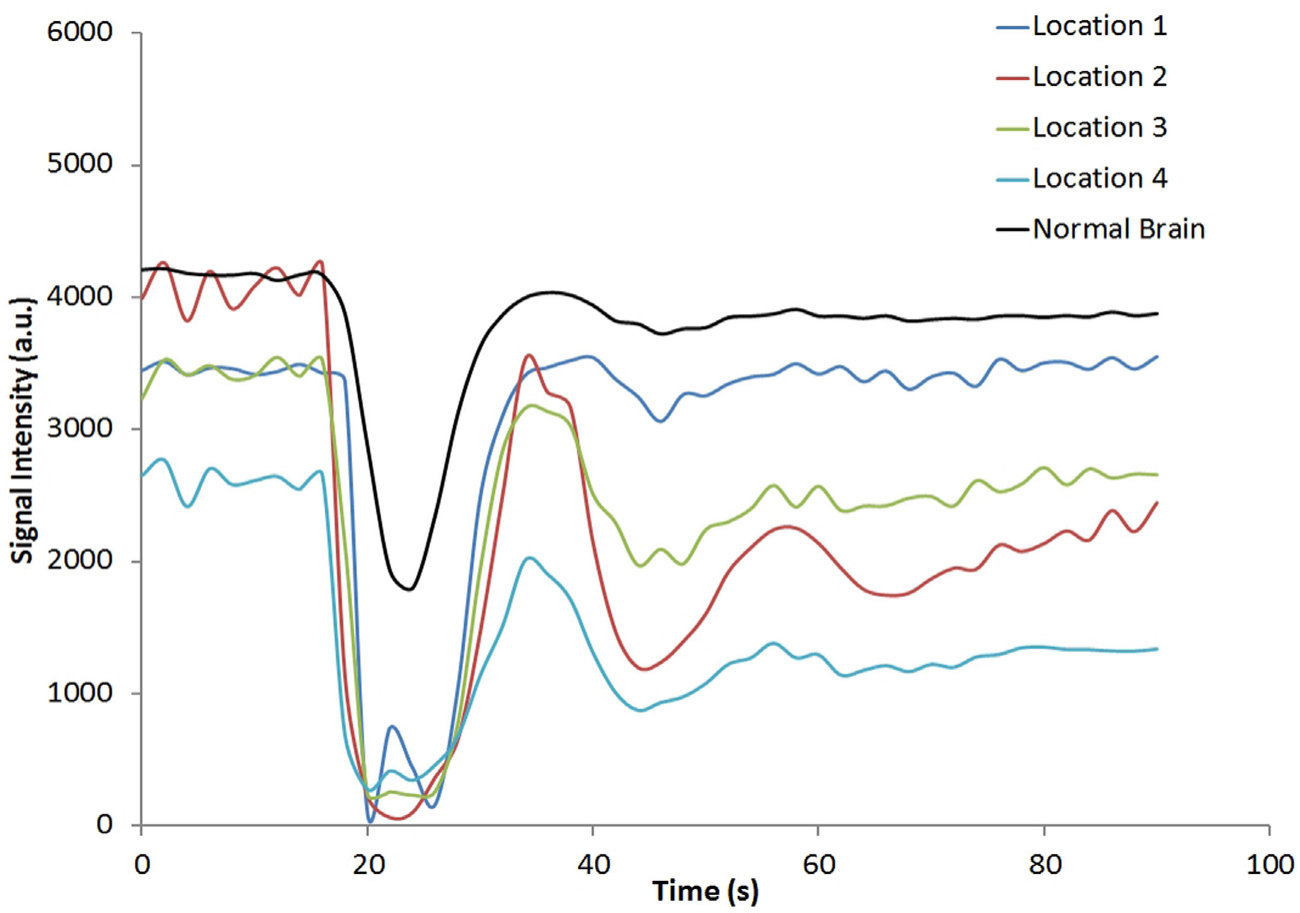
**Signal intensity time courses of different high blood volume locations and in normal brain tissue**. All locations except Location 2 have signal truncation artifacts due to high blood volume. Their respective locations are indicated by arrows in Figure 1.

**Figure 3:**
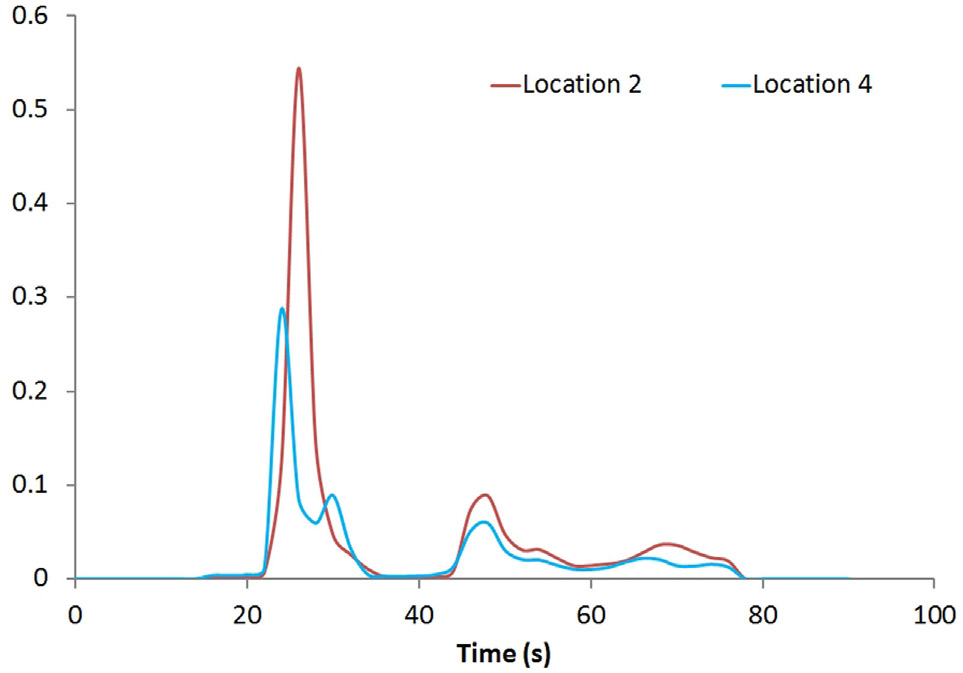
**Tissue residue functions at Locations 2 and 4**. Both the first-pass [20s, 40s] and second-pass [40s, 60s] of the function are well separated.

Figure 1 shows the rCBV and rCBF maps from the first-pass and second-pass tissue residue function of a representative 3 T case done with a power injector. Note the close resemblance of the rCBV and rCBF map generated with the second-pass tissue residue function, indicating a tight coupling. Figure 2 shows the truncation artifacts on the single-pixel signal time course within the time window of [16 s, 34 s] when the contrast bolus pass through different pixel locations. The mean noise level was about 100 au. Various forms of signal truncation are shown at Locations 1, 3, and 4, while Location 2 is not truncated. The truncation issue is very common in areas with high blood volume in tumor tissue, especially near blood vessels. Figure 3 shows the effect of signal truncation on the tissue residue function. The first-pass tissue residue function is essentially truncated near the top, which will significantly underestimate rCBF. The first-pass and second-pass portion of the tissue residue function are well separated, and the second-pass portion of the tissue residue function has a monotonic decay shape after the peak.

**Figure 4:**
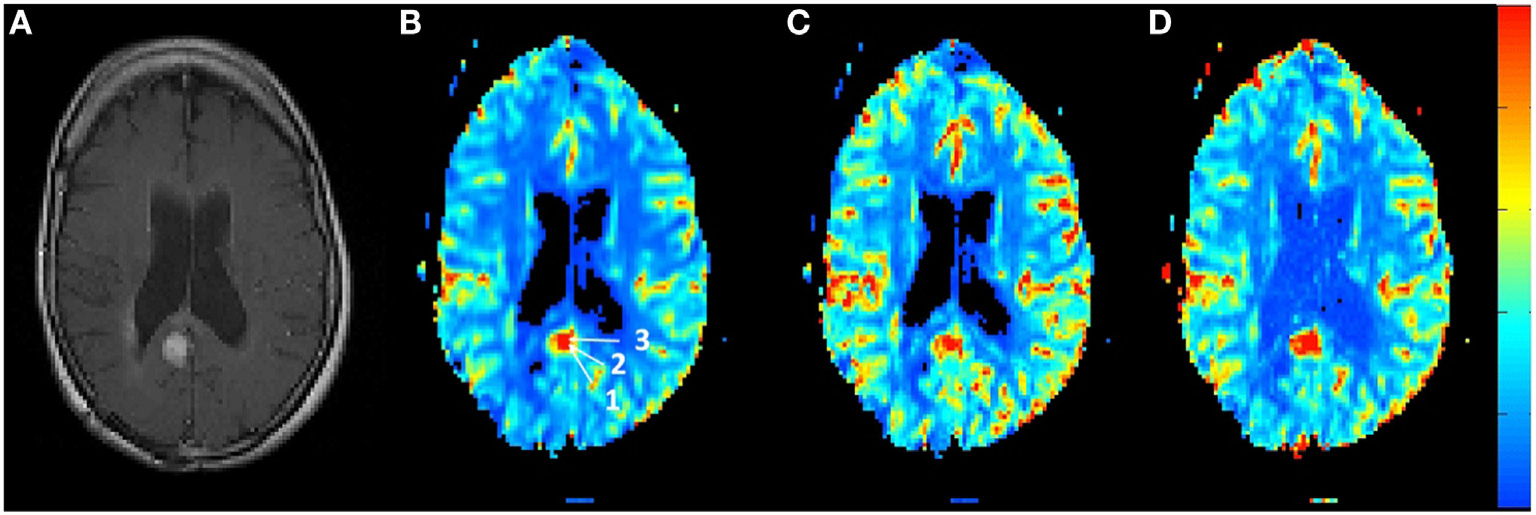
**(A)** T1-weighted post-contrast MRI showing the location of suspected tumor. The suspected enhanced region has a hot spot area in the **(B)** relative blood volume map as well as in the **(C)** relative cerebral blood flow map determined by the peak of the first-pass tissue residue function as well from the **(D)** second-pass tissue residue function.

**Figure 5:**
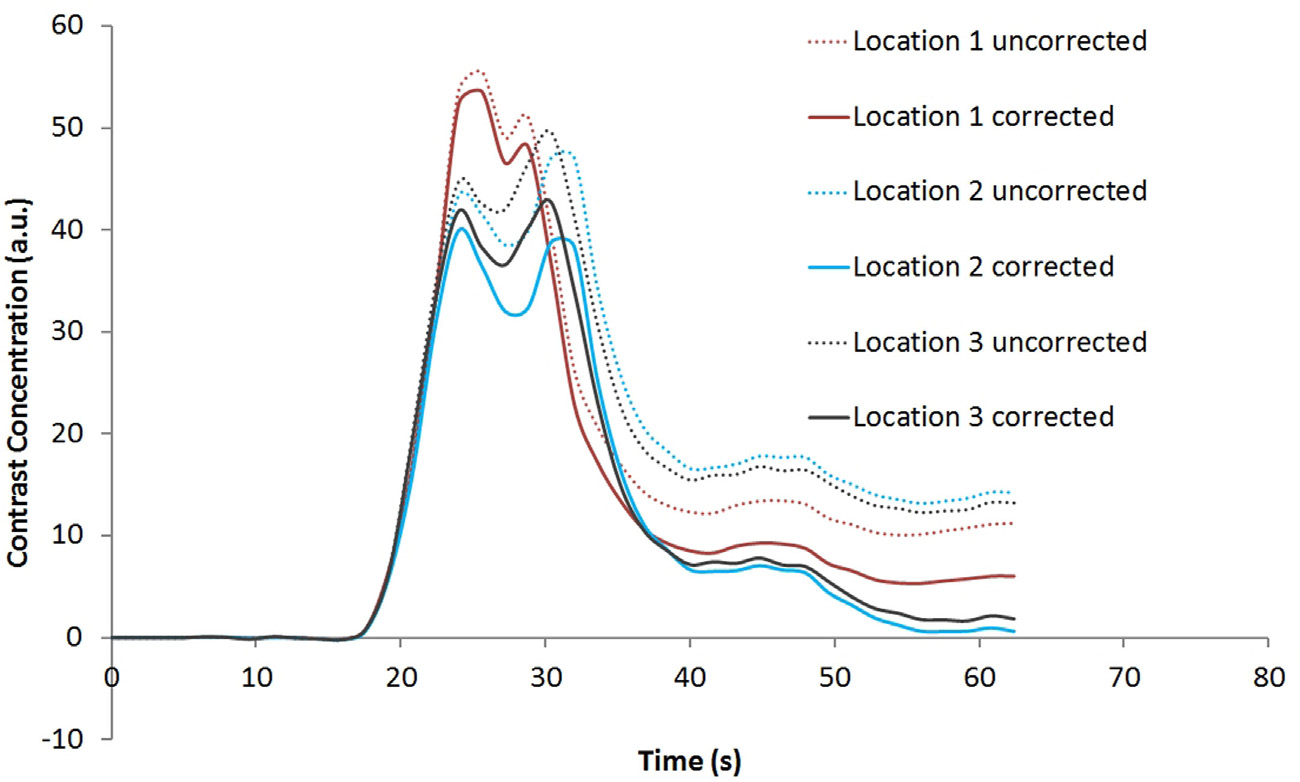
**Contrast concentration time courses of Locations 1–3 indicated in Figure 4B before and after contrast leakage correction**. Signal truncation artifact at these locations results in a disruption of the contrast concentration time course. The effect of leakage correction is obvious with minimal contrast concentration after the first and second passes of the bolus.

Figure 4 shows the rCBV and rCBF maps from the first-pass and second-pass tissue residue function of a representative 1.5 T case done without using a power injector. The post-contrast T1-enhanced region overlapped spatially with high rCBV and rCBF regions either using the peak of the first-pass or the second-pass tissue residue function. This is the worst-case scenario where manual injection was not fast enough to allow a clear separation between first-pass and second-pass bolus passage. It also violates the assumptions of a separable second-pass. Nevertheless, Figure 5 shows the effect of signal truncation and the eventual distortion on the contrast concentration time courses. The leakage correction still performs fine as the contrast concentration returns to very low level at steady-state in the presence of small signal truncation error.

## Discussion

One factor that limits our ability to quantify CBF, CBV, and MTT from DSC perfusion studies is the necessity of measuring the signal in the artery (i.e., AIF) that feeds the tissue of interest, whose shape depends on injection speed, cardiac output, etc. It has been shown that errors in selecting the AIF can be among the largest sources of error in the derived measurements of CBV, CBF, and MTT. Truncation of the AIF shape has a profound effect on the maps generated.

Truncation of the tissue signal time course also has a profound effect on the maps generated. This problem is less addressed in the literature as the effect is quite different on a pixel-to-pixel basis depending on the severity of truncation. The various shapes and form of truncation can be hard to recognize as saturation of contrast agent in part of a pixel is not equivalent to signal hitting the floor as partial volume effect determines the ultimate signal behavior. Image pixels with a large blood-containing fraction are more likely to have signal truncation artifacts. Truncation in the AIF can be mitigated to some extent by fitting the first-pass portion of the AIF using the gamma-variate function (17), which is also used in this study. Truncation in the tissue level is, however, difficult to compensate as the tissue residue function is unknown, and it can greatly affect the shape and form of the contrast concentration curves.

The proposed extension to the LAD deconvolution method works well in brain tumor cases that are based on the assumption that there is little overlap between the first-pass and second-pass portions of the tissue residue function. It applies generally where the contrast bolus is injected quickly with the help of a power injector where the first-pass and second-pass contrast passage are well separated. In tissue ischemia, where the MTT is very long, i.e., the tissue residue function has a long tail, there will be significant overlaps between the first-pass and second-pass portion. This limits application in tissue with known long transit time, such as choroid plexus, or in ischemic tissue. Note that this assumption is implicitly used in leakage correction, which models that the contrast concentration time course in tumor tissue is proportional to the contrast concentration time course of normal tissue. Therefore, leakage correction should be used and interpreted with caution near tissue with long transit time in brain tumor cases.

In addition, we would like to point out that, in LAD deconvolution of tissue residue function, temporal signal-to-noise ratio and the temporal contrast change during bolus passage are two important factors for the method to work properly rather than static image signal-to-noise ratios. To quantify the tissue residue function in more detail, one would need higher temporal resolution while simultaneously addressing the accompanying T1 leakage effects in tumor cases (18). Table 1 shows a summary of the technical pitfalls and potential solutions discussed in this paper.

**Table 1:** **Summary of technical pitfalls and potential solutions**.

Pitfalls	Solutions
Arterial input function truncation (AIF)	Fitting AIF with a Gamma-variate function
Brain tissue signal time course truncation	1. Leakage correction
	2. Calculate rCBF and rCBV using second-pass tissue residue function

One notable effect of using the second-pass tissue residue function in determining rCBF is that the major veins are suppressed. This is obvious in the suppression of sagittal sinus in Figure 1C compared to Figure 1B. This is understandable as major veins effectively collect the blood after the passage of the bolus through the whole brain and have significantly higher vascular dispersion. Sagittal sinus is the most suppressed. It is also noteworthy that, in the calculation of rCBV, signal truncation artifacts in major veins will also cause a truncation in the contrast concentration time curve, leading to underestimation of rCBV. This may have significant implications in tumor rCBV interpretation. The underestimation of rCBV due to signal truncation is less severe than the case in rCBF. It has been shown that rCBV and rCBF are two of the most important parameters to differentiate pseudoprogression and tumor recurrence in glioblastoma patients, after chemoradiation (2, 4–6). Radiation necrosis is associated with low rCBV and rCBF. Gross underestimation of rCBV and rCBF due to signal truncations would bias the diagnosis toward pseudoprogression, systematically. However, perfusion change during immunotherapy in glioblastoma could be therapy–specific, and more studies are needed to understand the role of perfusion imaging to monitor disease progression.

To summarize, we propose the use of second-pass tissue residue function in rCBF calculation using LAD deconvolution method. This strategy is used to circumvent the practical limitations in DSC PWI, where signal truncation artifacts occur often in high blood volume regions. The issue has not received much attention in the literature, and the problem is more severe in high-grade brain tumor where the rCBV is very high. Implications of signal truncation in high blood volume regions leading to underestimation in rCBV and rCBF are discussed. In the absence of severe tissue ischemia, rCBF maps derived from second-pass tissue residue function may be a useful alternative. By paying attention to the technical pitfalls described in this paper, rCBV and rCBF underestimations can be avoided. Unbiased estimations of rCBF and rCBV are crucial to differentiate pseudoprogression from tumor recurrence.

## Author Contributions

KW designed, conducted the studies, and prepared the manuscript. SF and PN provided clinical data and contributed to manuscript preparation. SW contributed to manuscript preparation.

## Conflict of Interest Statement

The authors declare that the research was conducted in the absence of any commercial or financial relationships that could be construed as a potential conflict of interest.
